# Editorial Comment: Impact of the advent of collagenase clostridium histolyticum on the surgical management of Peyronie's disease: a population-based analysis

**DOI:** 10.1590/S1677-5538.IBJU.2020.03.05

**Published:** 2020-02-20

**Authors:** S Sukumar, DB Pijush, S Brandes, Valter Javaroni

**Affiliations:** 1 Columbia University Department of Urology New YorkNY USA Department of Urology, Columbia University, New York, NY, USA; 2 Columbia University Department of Urology Department of Biostatistics New YorkNY USA Department of Urology, Department of Biostatistics, Columbia University, New York, NY, USA; 3 Columbia University Medical Center Reconstructive Urology New YorkNY USA Chief, Reconstructive Urology, Given Foundation Professor of Urology, Columbia University Medical Center, New York, NY, USA; 1 Hospital Federal do Andaraí Rio de Janeiro Departamento de Andrologia Rio de JaneiroRJ Brasil Departamento de Andrologia, Hospital Federal do Andaraí Rio de Janeiro, Rio de Janeiro, RJ, Brasil

## COMMENT

After the 2013 FDA approval of collagenase clostridium histolyticum (CCH) what was its impact on the use of surgical management of Peyronie Disease (PD) in United State? Dr. Sukumar and cols. from Columbia University hypothesized that with the introduction of CCH, surgery as a primary treatment modality for PD would be used less often.

The authors reviewed 547 men with PD registered in Statewide Planning and Research Cooperative System (SPARCS) that provides data on patients in the outpatient, inpatient, ambulatory, and emergency department setting in New York. All patients >18 years old with a diagnosis with PD who received surgical therapy (ST), defined as plaque excision/incision and grafting or plication, or injection therapy (IT) as a primary treatment between 2003 and 2016 were included.

Over the study period, surgical management was used less often as the primary procedure with a concurrent increase in use of IT (P < .001). On multivariable modeling, patients more likely to receive IT as treatment for penile curvature were younger, of higher socioeconomic status and presented to a surgeon with a high volume practice.

That trend should worry other countries were CCH could be approved? IMPRESS I and II data revealed that men treated with CCH showed a mean 34% improvement in penile curvature, representing a mean −9.3 ± 13.6 degree change per subject (p <0.0001) (1) after eight injections.

It is also relevant the many changes in CCH original protocol were proposed since its launch. The combination of traction devices and CCH, for example, seems to be associated with significantly greater curvature and length improvements compared with CCH alone (2).

Finally, in the first cost-effectiveness comparison of treatment modalities for PD (CCH, traction device and surgery) where the success was defined as ≥20% improvement in curvature, CCH was (by far) the most expensive and was not the most effective (3).
